# Intra-Articular Entrapment of the Medial Epicondyle following a Traumatic Fracture Dislocation of the Elbow in an Adult

**DOI:** 10.1155/2018/5401634

**Published:** 2018-01-31

**Authors:** Hicham G. Abdel Nour, George S. El Rassi, Jack C. Daoud, Youssef G. Hassan, Rami A. Ayoubi, Nabih I. Joukhadar

**Affiliations:** Department of Orthopedic Surgery and Traumatology, Saint Georges University Medical Center, Balamand University, P.O. Box 166378, Achrafieh, Beirut 1100 2807, Lebanon

## Abstract

Medial epicondyle entrapment after an acute fracture dislocation of the elbow is a common finding in the pediatric population, but a rare finding in adults. We present a case of an adult patient diagnosed with a traumatic fracture dislocation of the elbow joint with intra-articular entrapment of the medial epicondyle. After initial evaluation, closed reduction was done. Stability testing after reduction showed an unstable joint; thus, open reduction and internal fixation was decided.

## 1. Introduction

Medial epicondyle entrapment after an acute fracture dislocation of the elbow is a common finding in the pediatric population, but a rare finding in adults. The medial epicondyle is the last ossification center to fuse in the distal humerus, making the simple avulsion fracture very unlikely to occur after closure of the epiphyseal line [1]. The medial epicondyle fragment avulses due to a traction force by the medial collateral ligament typically by increased valgus stress or frequently an elbow dislocation [2]. A complex elbow instability posttraumatic elbow dislocation is a challenging entity to deal with and may have unsatisfactory results if not treated adequately [3]. We report here a case of an adult patient presenting with a traumatic posterolateral fracture dislocation of the left elbow with intra-articular entrapment of the medial epicondyle fragment.

## 2. Case

**Figure 1:**
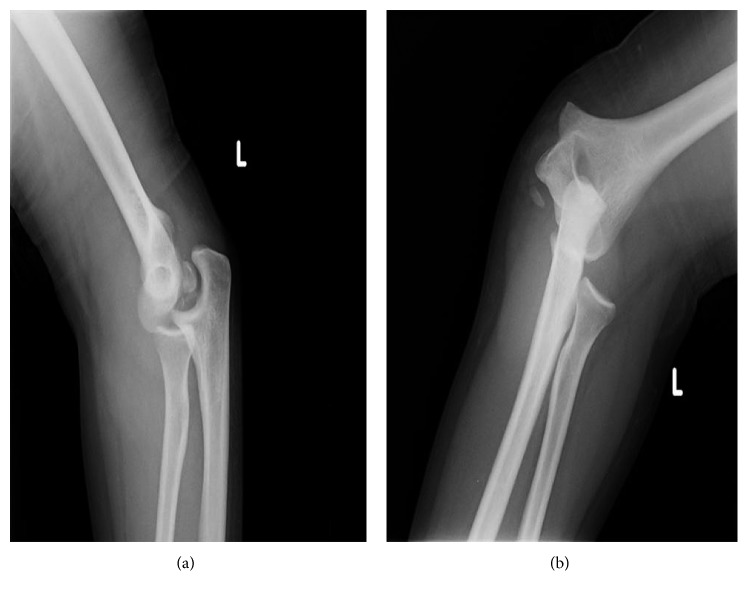
Lateral and anteroposterior radiographs of the left elbow joint showing posterolateral fracture dislocation with intra-articular entrapment of the avulsed medial epicondyle fragment. True lateral and anteroposterior radiographs could not be obtained due to the pain and the dislocation.

A 24-year-old male patient presented to our emergency department with severe left elbow pain and limited range of motion after falling from height on an outstretched hand 1 hour prior to his presentation. To note, the patient has no history of prior elbow dislocation or trauma during his childhood. On clinical examination, the patient was found to have posterolateral dislocation of his left elbow joint with severe tenderness over the medial aspect. There was minimal numbness over his left 4th and 5th fingers, but no other vascular involvement was noted. Anteroposterior and lateral radiographs of the left elbow were done that showed posterolateral dislocation of the left elbow joint with concomitant medial epicondyle avulsion fracture with intra-articular entrapment of that fragment (Figure 1).

**Figure 2:**
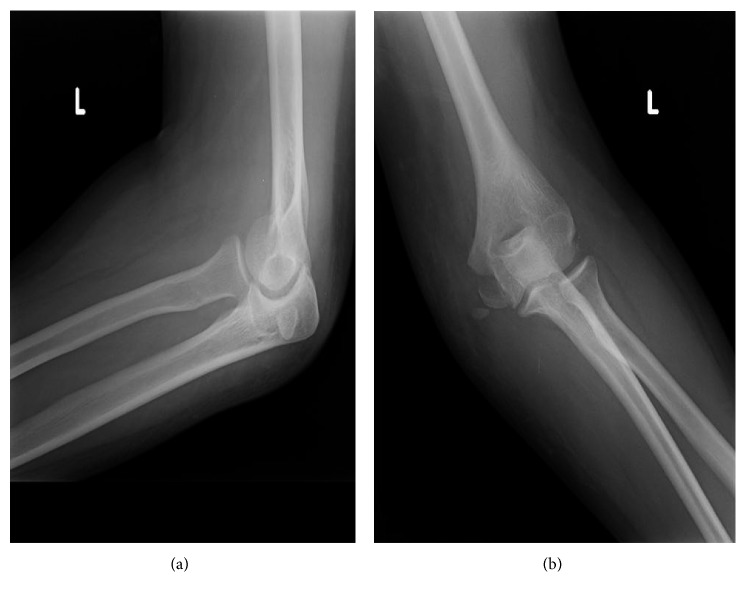
Anteroposterior and lateral radiographs of the left elbow joint after closed reduction showing a displaced medial epicondyle avulsion fracture.

Closed reduction was done under sedation, and after multiple failed attempts due to the entrapped medial epicondyle fragment, subsequent reduction of the medial epicondyle fragment was achieved (Figure 2).

**Figure 3:**
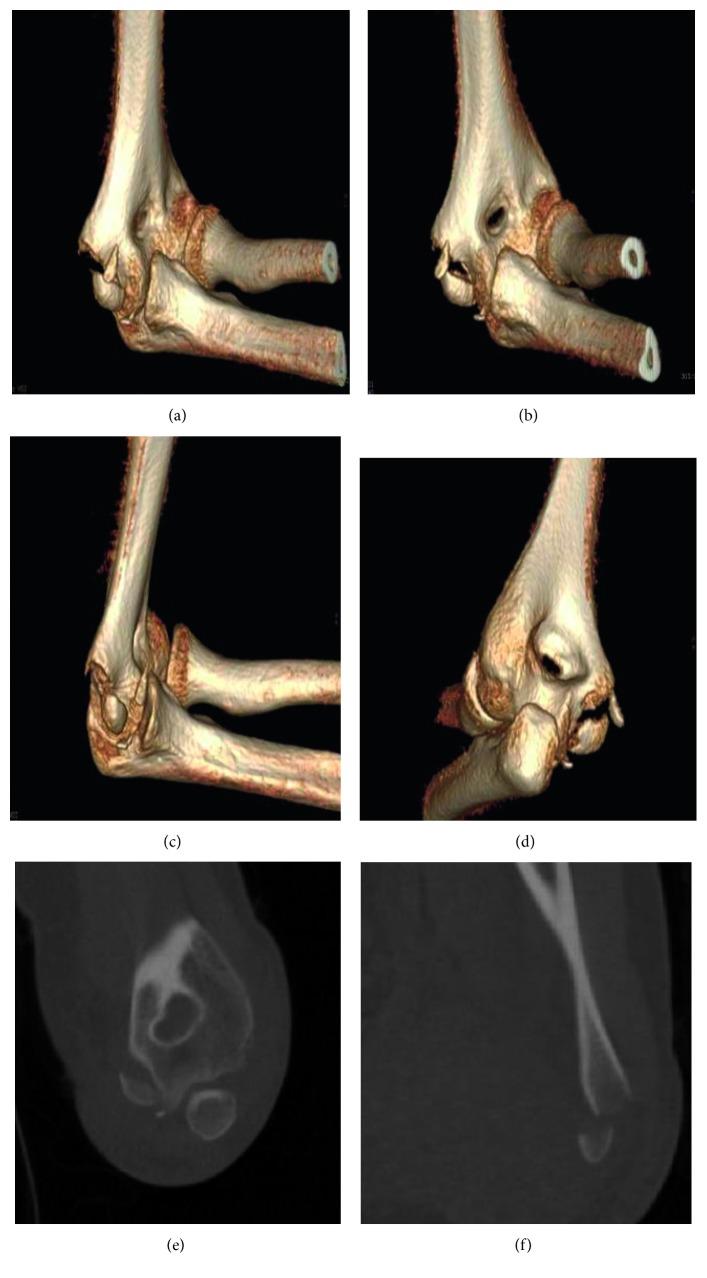
CT scan with 3D reconstruction after closed reduction showing a displaced medial epicondyle avulsion fracture with no other associated bony injury.

After the reduction, and for proper postreduction evaluation, varus and valgus stress tests under fluoroscopy were done that showed grand instability of the elbow joint. A computed tomography scan with 3D reconstruction was then ordered to rule out any associated missed fracture on radiograph and for adequate preoperative planning. The CT scan showed a displaced medial epicondyle avulsion fracture but no other associated bony injuries (Figure 3).

**Figure 4:**
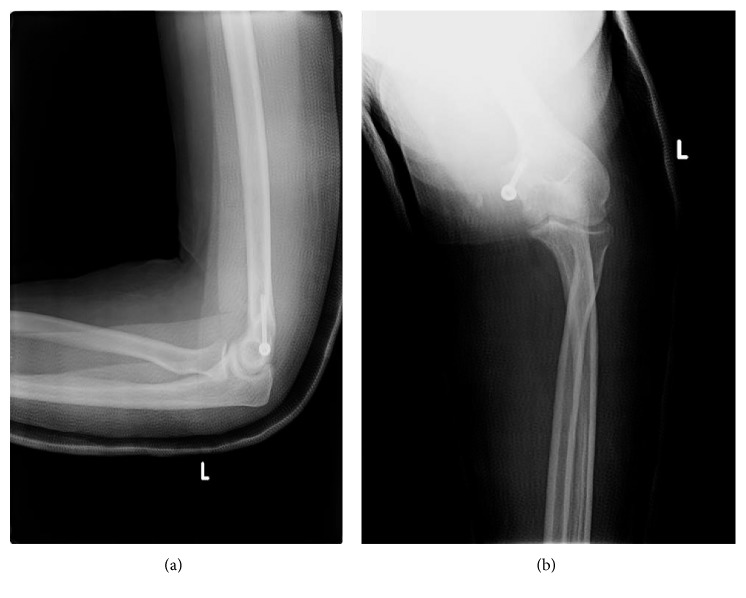
Postoperative control X-rays showing good reduction and compression of the medial epicondyle fragment. To note, the patient has an above-the-elbow posterior cast splint.

The next day, the patient was transferred to the operating theater for open reduction and internal fixation. In the operating room, with the patient placed in the supine position, general anesthesia and prophylactic antibiotics were administered. With his left upper limb on a hand table, a medial approach to the left elbow joint was done with a longitudinal 5 cm incision over the medial epicondyle. After appropriate dissection, the medial epicondyle fragment was identified, still partially entrapped in the ulnohumeral joint line, with its unruptured intact medial collateral ligamentous complex. The fragment was reduced with 1 K wire and fixed with 1 cannulated half-threaded 40 mm × 4.5 mm screw and a washer (Figure 4).

**Figure 5:**
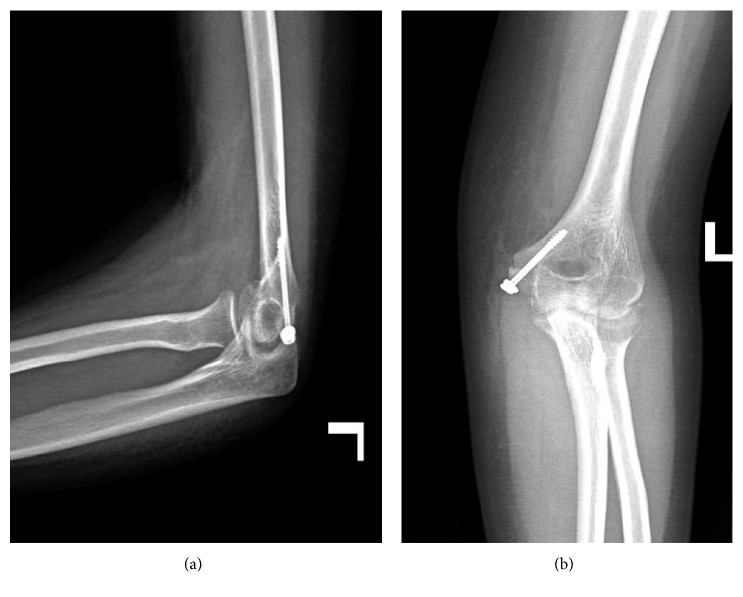
Left elbow control X-rays done at 3 weeks postop that showed proper integrity of the reduction.

A washer was used in order to optimize compression and not taking the risk of unintentional intrusion of the screw head through the cortical bone or fracturing the fragment. Careful inspection was done, leaving no entrapped soft tissue inside the fracture site during reduction. Postoperatively an above-the-elbow posterior splint was applied to the elbow joint in 90° of flexion with neutral forearm rotation. After 1 week, making sure that the swelling decreased and the 4th and 5th finger paresthesia has resolved, a full-circular above-the-elbow cast was applied in the same previous anatomic position. Control X-rays were done at 3 weeks postop that showed proper integrity of the reduction (Figure 5).

**Figure 6:**
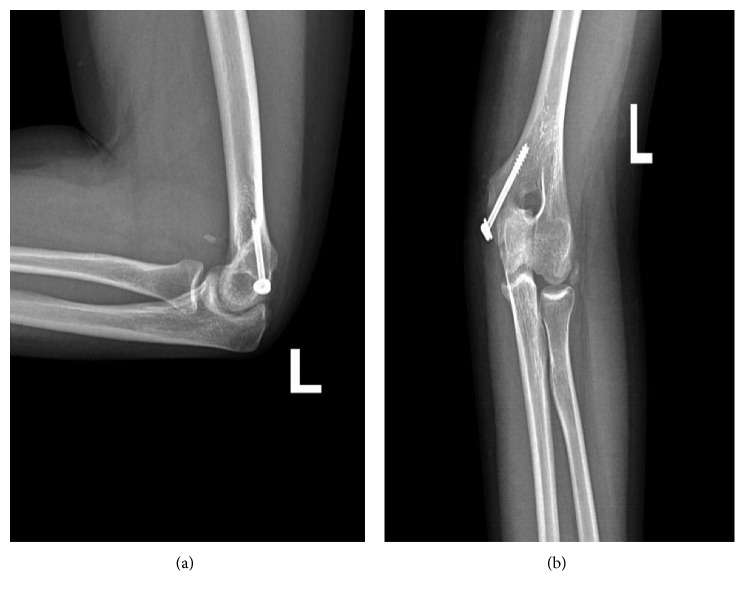
Lateral and anteroposterior radiographs of the left elbow joint 12 weeks postop showing union of the avulsed medial epicondyle fragment. Anterior and lateral ossifications seen indicate possible partial ligamentous injury that calcified with time and were not seen on previous radiographs.

So the decision was taken to remove the cast and keep the right arm in an arm sling for 1 more week with minimal careful range of motion. After that, physiotherapy was started consisting only of passive progressive flexion/extension range of motion with no varus or valgus stress for the following 3 weeks. At 12 weeks postop, the patient had full range of motion of his elbow joint and adequate varus and valgus stability, and control X-rays done showed union of the avulsed medial epicondyle fragment (Figure 6). At this point, the patient was allowed to get back to normal daily activities and will be allowed to resume sports activities at 6 months postoperatively. The future plan was removal of the screw at 6 months after the surgery.

## 3. Discussion

Medial epicondyle fractures are commonly described fractures in the pediatric age groups, as its epiphyseal line is the last ossification center to fuse in the distal humerus between ages 15 and 20 [1, 2]. Medial epicondyle fractures present most commonly posttraumatic pediatric elbow dislocation, and in 15% to 25% of these cases, the medial epicondyle becomes entrapped in the ulnohumeral joint. If the medial epicondyle fragment is seen at the level of the joint line after closed reduction of a traumatic elbow dislocation, partial entrapment should at least be considered [1]. This entity is rarely described throughout the literature in adults.

Purser mentioned two cases of adult fracture dislocation of the elbow with intra-articular inclusion of the medial epicondyle fragment. One of them was treated with open reduction but no fixation after multiple failed trials of closed reduction. The second case was a nonunion of a medial epicondyle fracture with recent posttraumatic intra-articular entrapment of that fragment. The latter patient was treated with closed reduction only. Purser concluded that in case the medial epicondyle fragment was really entrapped, then the affected joint would re-dislocate after full extension after closed reduction [4].

Khan and Zahid [2] reported two adult cases of fracture dislocation of the elbow with intra-articular inclusion of the medial epicondyle fragment. One case was treated with closed reduction, and the other required open reduction and fixation with 2 K wires. They noted one case with redisclocation after full extension trial after closed reduction.

Rodriguez-Martin et al. [3] reported a case of a posttraumatic medial fracture dislocation of the elbow with a medial epicondyle displaced fracture but with an associated coronoid process fracture and severe soft tissue injury.

In a retrospective study done in a hospital in Montpellier, France, Louahem et al. [5] reported 130 pediatric cases with displaced medial epicondyle fractures. All cases were treated with open reduction and internal fixation with either K wires or compression screws. They concluded that operative fixation is a necessity in such fractures to ensure proper union and prevent valgus instability.

In our case, testing after closed reduction showed only valgus and varus instability, but no redislocation was noted in full extension. A preoperative CT scan for evaluating any associated missed fracture on radiograph is not always mandatory. An MRI in this case could have been helpful for evaluating any associated ligamentous injuries. Decision was taken to perform an open reduction and internal fixation with a compression screw and a washer to ensure proper reduction, subsequent union with minimal risk of unintentional screw head intrusion into the fragment, and ensure early range of motion to prevent elbow stiffness. Keeping the cast for 4 weeks was not obligatory but was done to guarantee proper healing of the ligaments in order to obtain good elbow stability afterwards even if it could take few more physiotherapy sessions to regain full range of motion.

## 4. Conclusion

Fracture of the medial epicondyle is a rare entity in adults but frequent in the pediatric age group as traction of the medial collateral ligament can easily cause an avulsion of the nonfused medial epicondyle [2]. In addition, associated intra-articular entrapment of the medial epicondyle after elbow dislocation is rarely mentioned in the reported cases throughout the literature. Careful preoperative planning, operative treatment, and postoperative management are essential and if not managed properly can frequently result in unsatisfactory outcomes.
